# Critical care nurses’ self-efficacy during the COVID-19 pandemic: a cross-sectional study

**DOI:** 10.3389/fpubh.2025.1557767

**Published:** 2025-06-11

**Authors:** Homood A. Alharbi

**Affiliations:** Medical–Surgical Department, College of Nursing, King Saud University, Riyadh, Saudi Arabia

**Keywords:** COVID-19, critical care, intensive care units, nurses, self-efficacy, Saudi Arabia

## Abstract

**Background:**

As frontline healthcare workers, nurses played major roles during the COVID-19 pandemic, especially in intensive care units (ICUs) where severe cases were managed. In this cross-sectional study, ICU nurses in Riyadh, Kingdom of Saudi Arabia, were surveyed to examine their self-efficacy during the pandemic. Hence, this study aimed to assess the level of SE among ICU nurses, and to explore its associations and differences based on specific sociodemographic and work-related factors.

**Methods:**

The data obtained from 135 ICU nurses using a self-administered validated General Self Efficacy (GSE) scale was subjected to the analysis of variance and Spearman coefficient Pearson correlation tests.

**Results:**

The self-efficacy score (31.8 ± 7.46) varied significantly according to age, nationality, and religion (at *p* = 0.04, 0.00, and 0.002, respectively). Older nurses tended to provide more favorable responses to the GSE items. Indian nurses exhibited the highest self-efficacy levels, followed by their Filipino counterparts, while Saudi nurses demonstrated the lowest levels.

**Conclusion:**

According to ICU nurses’ self-reported GSE scale responses, they demonstrated a high level of self-efficacy during the pandemic. While these findings are based on a small and relatively homogenous cohort recruited at a single healthcare institution, they suggest that greater access to training and mentorship will enable Saudi ICU nurses to confidently and efficiently take increasingly independent and complex clinical roles.

## 1 Introduction

The coronavirus disease 2019 (COVID-19) is a global pandemic caused by the severe acute respiratory syndrome coronavirus 2 virus (SARS-CoV-2) with varying clinical presentations (1, 2). While 80% of cases are mild, 20% develop severe illness, and 5% become critically ill, requiring intensive care, mechanical ventilation, or treatment for complications like pneumonia and acute respiratory distress syndrome (2, 3). Other complications which can be fatal include septic shock, metabolic acidosis, and coagulation disorders (4). Patients experiencing these complications are classified as high-risk individuals often presenting with associated co-morbidities such as diabetes, hypertension, cardiovascular and respiratory diseases, kidney disorders, malignancies, and HIV (4). During the COVID–19 pandemic (5–7), most nations struggled to deliver care to all those that required it, but this issue was particularly acute in the Kingdom of Saudi Arabia (KSA) due to one of the highest infection rates in the Middle East (8). By June 9th, 2024, there were more than 775 million confirmed cases of the disease across the globe, resulting in over seven million fatalities, even though 5.47 billion vaccine doses had been distributed worldwide (7). By October 20th, 2024, 841,469 cases had been documented in the KSA since the beginning of the pandemic, the second highest number among the six Arab Gulf nations (7). While the number of COVID-19 patients has subsided since the pandemic peak, as most non-urgent care had to be scaled down to cope with COVID-19 cases, several hospitals in the country, including the King Saud University Medical City (KSUMC), are still working to return to the normal mode of operation.

In addition, as frontline healthcare workers (HCWs), especially nurses, are at high-risk of nosocomial infection (9), they play a key part in direct, high-quality patient care (10). As noted by the American Psychiatric Nurses Association (11), during the pandemic, nurses experienced immense “pressure, fear, exhaustion, isolation and ongoing emotional trauma” which had adverse effect on their “mental health, safety, and ability to provide the best possible care.” Due to these challenges nurses confronted during the pandemic (12), their perceived self-efficacy may decline, affecting how they behave, think, and feel about their capacity to deal with similar circumstances in the future (13). According to Bandura (14), self-efficacy refers to “an overall self-confidence that an individual respond to different environmental challenges or face new things.” Given ample evidence that low self-efficacy contributes to anxiety, depression, and stress (15–17), increasing it is crucial for mental well-being and ability to cope with stress (17–19).

Although nurses’ self-efficacy during the COVID-19 pandemic has been extensively studied (10, 20–22), particularly among ICU nurses (23, 24), there is limited data on the Arabian Gulf region, with only one investigation pertaining to the KSA (25) and another to nearby Oman (26). As neither study was conducted in a tertiary care university setting, the research presented here aims to fill that gap by assessing the self-efficacy of practicing registered nurses that worked in intensive care units (ICUs) in a university medical city in the KSA during the COVID-19 pandemic. This study specifically highlights a research gap concerning the limited data on nurses’ self-efficacy during the COVID-19 pandemic in the Arab Gulf countries, particularly in Saudi Arabia. While numerous studies have been conducted globally, only one prior study has explored this topic in Saudi Arabia and another in Oman. However, neither was conducted in a tertiary care university setting. This study aims to bridge this gap by investigating the self-efficacy of registered nurses working in the ICUs within a university medical city in the KSA during the pandemic.

## 2 Materials and methods

### 2.1 Study design

This quantitative study was based on a cross-sectional, correlational design.

### 2.2 Sample and setting

Using convenience sampling, the respondents were recruited from the pool of licensed practicing nurses that worked at the KSUMC (a tertiary referral and teaching healthcare institution inside in King Saud University in Riyadh) between September and October 2020 and had extensive experience in treating COVID-19 patients. The hospital ranks among the largest healthcare institutions in the nation, specifically addressing the medical management of patients affected by COVID-19.

All ICU nurses employed at KSUMC were invited to participate. The ICU nurses were eligible to participate if they were currently practicing as registered nurses, possessed a valid nursing qualification and license, had at least one year of professional experience, were proficient in the English language, and provided informed consent to participate in the study. The study excluded registered nurses who were working in intermediate care (step-down) units, emergency unit, burn unit and medical-surgical units, and those with less than one year of professional experience in the ICUs.

The sample size was calculated using G*Power version 3.1.9.7 software, which is designed for A-priori power analysis necessary for conducting a statistical test of analysis of variance (ANOVA). The power analysis suggested that to perform ANOVA across three groups, a minimum sample size of 135 is sufficient to attain a medium effect size of 0.25, an alpha level at 0.05, and a power of 0.95.

### 2.3 Ethical consideration

The Institutional Review Board (IRB) of the College of Medicine in KSUMC reviewed and approved the study (Ref. No. E-20-4833). This research adhered to the ethical standards pertinent to survey studies, emphasizing the principles of justice, informed consent, and respect for human dignity. The principle of justice encompassed a thorough explanation of the study’s risks and benefits, an acknowledgment of the respondents’ cultural backgrounds, the assurance of confidentiality regarding the collected data, and respect for the respondents’ right to privacy. Throughout the study, respondents were not subjected to any mental, emotional, or other forms of harm. Informed consent was secured after providing the respondents with comprehensive and truthful information about the study, along with the opportunity to pose questions. Respondents were also made aware that their involvement was entirely voluntary and that they could withdraw from the study at any time without facing any adverse repercussions.

### 2.4 Instrument

The data for this study was obtained via close-ended self-administered questionnaire inquiring into respondents’ sociodemographic (age, gender, marital status, nationality, religion, education level, length of service as a nurse and as an ICU nurse, and ICU sub-unit) and work-related (information on care provided to patients with suspected and confirmed COVID-19 infection) data, as well as general self-efficacy (GSE). For GSE assessment, a validated 10-item self-efficacy scale (Cronbach’s alpha = 0.76–0.90) was used (27), whereby responses were provided on a 4-point Likert scale where 1 = not at all true, 2 = hardly true, 3 = moderately true, and 4 = exactly true (27–29). Accordingly, possible scores ranged from 10 to 40, with higher scores signifying greater self-efficacy. To frame the scale items in relation to the nurses’ experience of COVID-19 pandemic, the following phrase preceded the 10 items: “Based on your experience in the clinical settings during the COVID-19 crisis, kindly provide your most honest response on the following items using the following options”.

### 2.5 Data collection

With support of their respective department managers, nurses who agreed to participate completed the survey during break times and were instructed to place the questionnaires in designated boxes placed at each ICU nurses’ station. Completed questionnaires were retrieved every Thursday and were kept in a secured cabinet.

### 2.6 Data analysis

The information obtained through the survey was analyzed using IBM SPSS version 27 (IBM Corp., Armonk, NY, United States). Sociodemographic variables and those related to work and self-efficacy were subjected to descriptive analyses (e.g., percentage, mean, and standard deviation). While Spearman coefficient Pearson correlation (*r*) was calculated to identify possible specific associations between study variables (age and experience). Spearman coefficient Pearson correlation test was used based on the findings from the normality tests, specifically the Kolmogorov–Smirnov and Shapiro–Wilk tests, which indicated that the data did not follow a normal distribution, as evidenced by *p*-values < 0.05 (*p*-values > 0.05 indicate normal distribution). Moreover, following the execution of the homogeneity of variance test, which yielded a *p*-value of 0.067, it was determined that the assumption of homogeneity of variance was met because the *p*-value is more than 0.05, thereby allowing for the subsequent calculation of the analysis of variance (ANOVA) to establish whether observed variations in self-efficacy differed depending on gender, qualifications, and experience. Statistical significance was set at *p* < 0.05.

## 3 Results

As can be seen from Table 1 summarizing their sociodemographic information, 129 (96%) of the respondents were women, 96 (71%) had a diploma in nursing, 127 (94%) were foreigners, 112 (83%) were married, and 102 (75%) were aged above 30. Their age ranged from 24 to 56 (*M* = 36.6, *SD* = 7.8) years. In terms of general experience as a nurse, 47.4% of the respondents selected 0–10 years, while 35.6% indicated 11–20 years. When asked about their ICU experience, 57.1% of the nurses selected 0–10 years, while 31.1% chose 11–20 years.

**Table 1:** Sociodemographic and work-related characteristics of nurses (*N* = 135).

#	Variables	Frequency (%)
1	Age (by years)	
	22 to 26	12 (9%)
	27 to 30	21 (15.6%)
	31 to 35	30 (22.2%)
	36 to 40	34 (25.2%)
	More than 40	38 (28%)
2	Gender	
	Male	6 (4%)
	Female	129 (96%)
3	Marital status	
	Married	112 (83%)
	Single	23 (17%)
4	Nationality	
	British	1 (0.7%)
	Filipino	28 (20.7%)
	Indian	86 (63.7%)
	Jordanian	9 (6.7%)
	Saudi	8 (5.9%)
	South African	3 (2.2%)
5	Religion	
	Christianity	96 (71%)
	Hinduism	18 (13.4%)
	Islam	21 (15.6%)
6	Education level	
	Doctorate or master’s level	2 (1.5%)
	Bachelor of science in nursing	37 (27.5%)
	Diploma in nursing	96 (71%)
7	Experience as a nurse	
	0 to 10	64 (47.4%)
	11 to 20	48 (35.6%)
	21 to 30	22 (16.3%)
	> 30	1 (0.7%)
8	Experience as ICU nurse	
	0 to 10	77 (57.1%)
	11 to 20	42 (31.1%)
	21 to 30	15 (11.1%)
	> 30	1 (0.7%)
9	Vaccinated against COVID-19	
	Yes	133 (90.5%)
	No	2 (9.5%)
10	Confirmed COVID-19	
	Yes	123 (91%)
	No	12 (9%)
11	Educational activities related to COVID-19	
	Yes	119 (88%)
	No	16 (12%)
12	ICU sub-unit	
	AICU	38 (28.1%)
	CCU	5 (3.7%)
	CICU	29 (21.5%)
	NICU	26 (19.3%)
	PICU	37 (27.4%)

AICU = Adult ICU; CCU = Coronary Care Unit; CICU = Cardiac ICU; NICU = Neonatal ICU; PICU = Pediatric ICU.

**Figure 1:**
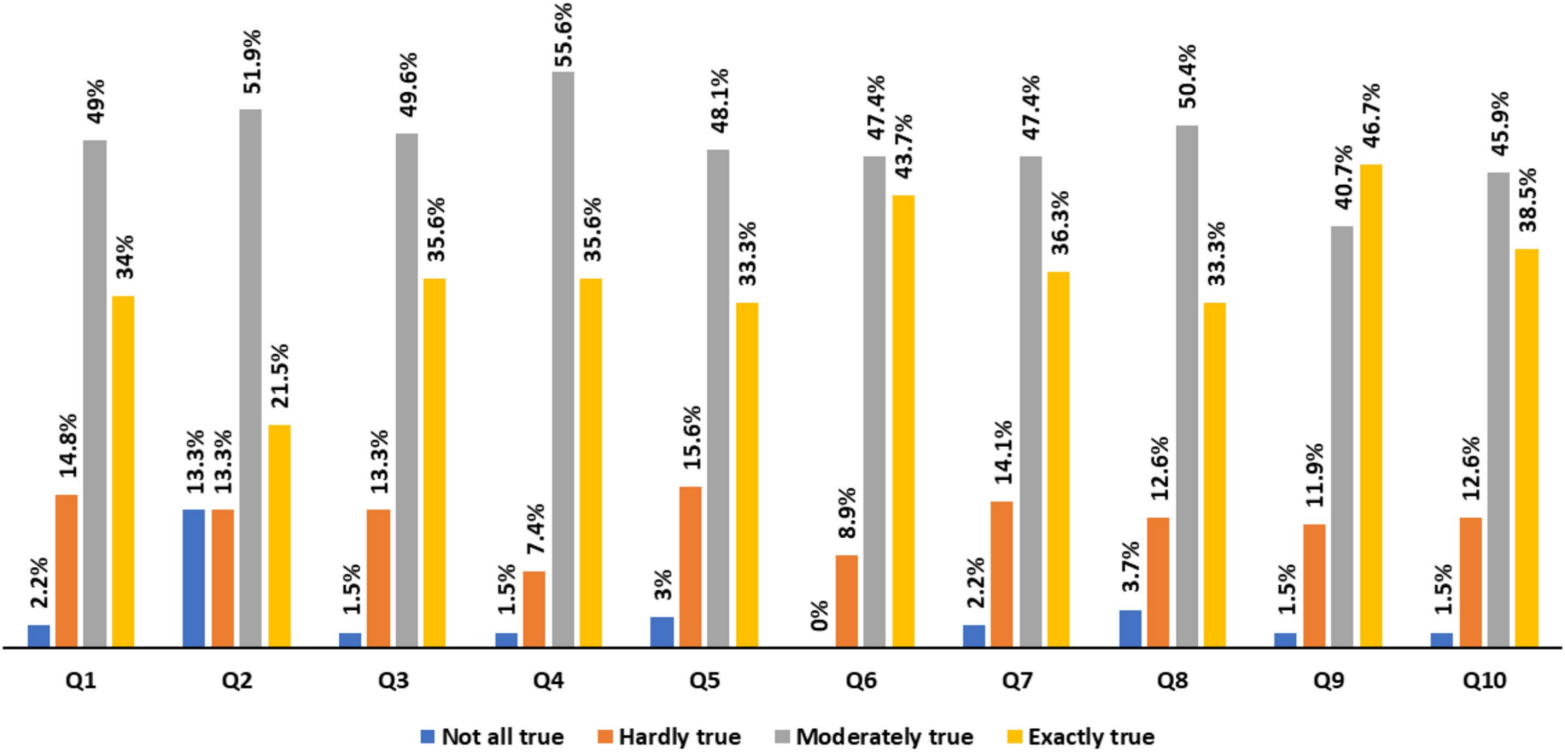
Self-efficacy items: response rate by nurses. Q1: I can always manage to solve difficult problems if I try hard enough, Q2: If someone opposes me, I can find the means and ways to get what I want. Q3: It is easy for me to stick to my aims and accomplish my goals. Q4: I am confident that I could deal efficiently with unexpected events. Q5: Thanks to my resourcefulness, I know how to handle unforeseen situations. Q6: I can solve most problems if I invest the necessary effort. Q7: I can remain calm when facing difficulties because I can rely on my coping abilities. Q8: When I am confronted with a problem, I can usually find several solutions. Q9: If I am in trouble, I can usually think of a solution. Q10: I can usually handle whatever comes my way.

As shown in Table 2 and Figure 1, their self-efficacy scores ranged from 19 to 40 (*M* = 31.8, *SD* = 7.46). The highest (3.4) score related to Question 6 (where none of the nurses chose “not at all true”), followed by Question 4 and Question 9 (3.3).

**Table 2:** Self-efficacy scores (mean and SD) according to scale items.

Items	Minimum	Maximum	Mean (SD)
Q1. I can always manage to solve difficult problems if I try hard enough.	1	4	3.2 (0.75)
Q2. If someone opposes me, I can find the means and ways to get what I want.	1	4	2.8 (0.92)
Q3. It is easy for me to stick to my aims and accomplish my goals.	1	4	3.2 (0.72)
Q4. I am confident that I could deal efficiently with unexpected events.	1	4	3.3 (0.66)
Q5. Thanks to my resourcefulness, I know how to handle unforeseen situations.	1	4	3.1 (0.77)
Q6. I can solve most problems if I invest the necessary effort.	2	4	3.4 (0.64)
Q7. I can remain calm when facing difficulties because I can rely on my coping abilities.	1	4	3.2 (0.75)
Q8. When I am confronted with a problem, I can usually find several solutions.	1	4	3.1 (0.77)
Q9. If I am in trouble, I can usually think of a solution.	1	4	3.3 (0.71)
Q10. I can usually handle whatever comes my way.	1	4	3.2 (0.77)
**Total**			**31.8 (7.46)**

Possible score range = 10–40; SD = Standard Deviation; 1 = Not all true, 2 = Hardly true, 3 = Moderately true, 4 = Exactly true.

Based on the results reported in Table 3, it is evident that age, nationality, and religion significantly affected self-efficacy (*p* = 0.04, 0.00, and 0.002, respectively). The age effect was particularly pronounced for nurses aged 36 and above. While South African nationality, followed by Indian and Filipino, was the most impactful, Hinduism was the religion most closely related to self-efficacy. On the other hand, SE did not vary significantly with the educational attainment, experience as a nurse/ICU nurse, ICU subunit, exposure to patients with confirmed COVID-19 infection, and participation in continuing medical education related to COVID-19. Although no significant relationship of vaccination status with self-efficacy was established, it is noteworthy that 90.5% of the nurses who were vaccinated against COVID-19 (Table 1) had a mean self-efficacy score of 31.7 (5.3) (Table 3).

**Table 3:** Self-efficacy by sociodemographic and work-related variables of nurses (*N* = 135).

Variables	Self-efficacy—mean (SD)	Self-efficacy – scores
	F (*P*-value)	Min–Max
Age (Y)		
22 to 26	28.4 (6.7)	19–40
27 to 30	30.9 (5)	23–40
31 to 35	30.8 (4.9)	21–40
36 to 40	32.5 (5.1)	21–40
> 40	33.2 (5)	21–40
	2.6 (0.04)*	
Gender		
Male	30 (7.8)	21–38
Female	31.8 (5.2)	19–40
	0.67 (0.42)	
Marital Status		
Married	31.8 (5.2)	21–40
Single	31.2 (5.9)	19–40
	0.29 (0.59)	
Nationality		
British	30 (−)	30–30
Filipino	30.9 (4.6)	21–39
Indian	32.7 (4.9)	21–40
Jordanian	30.6 (4.5)	24–37
Saudi	24 (6.3)	19–38
South African	36.7 (0.58)	36–37
	5.6 (0.00)*	
Religion		
Christianity	32.2 (4.9)	21–40
Hinduism	33.4 (5.1)	24–40
Islam	28.1 (5.9)	19–38
	6.8 (0.002)*	
Education level		
Doctorate or master’s	35.5 (6.4)	31–40
Bachelor	32.1 (5.5)	21–40
Diploma	31.5 (5.2)	19–40
	0.68 (0.51)	
Experience as a nurse		
0 to 10	30.8 (5.5)	19–40
11 to 20	32.2 (5)	21–40
21 to 30	33 (5)	21–40
> 30	38 (−)	38–38
	1.7 (0.17)	
Experience as ICU nurse		
0 to 10	31.2 (5.3)	19–40
11 to 20	32.2 (5.1)	21–40
21 to 30	32.6 (5.7)	21–40
> 30	38 (−)	38–38
	0.94 (0.42)	
Vaccinated against COVID-19		
Yes	31.7 (5.3)	19–40
No	35.5 (3.5)	33–38
	1.04 (0.31)	
Confirmed COVID-19		
Yes	31.9 (5.2)	19–40
No	29.9 (5.7)	21–40
	1.5 (0.22)	
Educational activities COVID-19		
Yes	31.9 (5.1)	21–40
No	30.1 (6.1)	19–40
	1.8 (0.18)	
ICU sub-unit		
AICU	32.5 (4.9)	21–40
CCU	33 (4.5)	28–39
CICU	30.7 (5.9)	19–40
NICU	32.7 (5.4)	22–40
PICU	30.8 (5.1)	21–40
	1.02 (0.40)	

AICU = Adult ICU; CCU = Coronary Care Unit; CICU = Cardiac ICU; NICU = Neonatal ICU; PICU = Pediatric ICU. * Significant at the 0.05 level (2-tailed).

According to the data reported in Table 4, age significantly affected responses to Question 4 (*p* = 0.047) and Question 6 (*p* = 0.008).

**Table 4:** Self-efficacy scale response, by age (years) of nurses (*N* = 135).

Items	From 22 to 26				From 27 to 30				From 31 to 35				From 36 to 40				More than 40				*p* value
	1	2	3	4	1	2	3	4	1	2	3	4	1	2	3	4	1	2	3	4	
Q1	1	2	7	2	0	5	10	6	1	3	19	7	0	7	16	11	1	3	14	20	0.20
Q2	3	2	3	4	1	3	14	3	6	5	13	6	4	4	20	6	4	4	20	10	0.65
Q3	0	4	6	2	1	2	10	8	0	4	17	9	1	4	19	10	0	4	15	19	0.39
Q4	0	4	6	2	0	1	15	5	1	1	18	10	0	3	19	12	1	1	17	19	0.047*
Q5	0	5	4	3	1	2	13	5	0	5	19	6	1	4	14	15	2	5	15	16	0.17
Q6	0	4	6	2	0	3	12	6	0	3	15	12	0	2	11	21	0	0	20	18	0.008*
Q7	1	2	7	2	0	5	10	6	1	6	15	8	0	3	16	15	1	3	16	18	0.43
Q8	2	1	6	3	1	4	12	4	2	4	18	6	0	3	17	14	0	5	15	18	0.12
Q9	0	5	5	2	0	3	7	11	1	3	13	13	0	2	15	17	0	3	15	20	0.13
Q10	0	2	6	4	1	4	9	7	0	4	16	10	1	3	17	13	2	4	14	18	0.92

1 = Not all true, 2 = Hardly true, 3 = Moderately true, 4 = Exactly true. * Significant at the 0.05 level (2-tailed).

Nationality and religion also played a role in the way nurses responded to most of the self-efficacy items (Tables 5, 6, respectively).

**Table 5:** Self-efficacy items by nationality of nurses (*N* = 135).

Items	British (*n* = 1)				Filipino (*n* = 28)				Indian (*n* = 86)				Jordanian (*n* = 9)				Saudi (*n* = 8)				South African (*n* = 3)				*P*-value
	1	2	3	4	1	2	3	4	1	2	3	4	1	2	3	4	1	2	3	4	1	2	3	4	
Q1	0	0	1	0	0	5	15	8	2	13	42	29	0	0	5	4	1	2	3	2	0	0	0	3	0.47
Q2	0	0	1	0	6	5	16	1	7	9	45	25	1	0	5	3	1	4	3	0	3	0	0	0	0.00*
Q3	0	0	1	0	0	2	17	9	1	10	39	36	1	1	7	0	0	5	3	0	0	0	0	3	0.001*
Q4	0	0	1	0	0	3	20	5	1	2	46	37	0	0	7	2	1	5	1	1	0	0	0	3	0.00*
Q5	0	0	1	0	0	5	15	8	2	11	43	30	1	1	5	2	1	4	1	2	0	0	0	3	0.097
Q6	0	0	1	0	0	3	15	10	0	4	39	43	0	1	7	1	0	4	2	2	0	0	0	3	0.001*
Q7	0	0	1	0	0	4	17	7	1	10	38	37	0	2	5	2	2	3	2	1	0	0	1	2	0.006*
Q8	0	0	1	0	1	4	15	8	0	11	44	31	1	0	6	2	3	2	2	1	0	0	0	3	0.00*
Q9	0	0	1	0	0	3	17	8	0	7	30	49	0	1	6	2	1	5	1	1	0	0	0	3	0.00*
Q10	0	0	1	0	0	1	18	9	3	11	36	36	1	0	5	3	0	5	2	1	0	0	0	3	0.04*

1 = Not all true, 2 = Hardly true, 3 = Moderately true, 4 = Exactly true. * Significant at the 0.05 level (2-tailed).

**Table 6:** Self-efficacy scale response, religion of nurses (*N* = 135).

Items	Christianity				Hinduism				Islam				*P* value
	1	2	3	4	1	2	3	4	1	2	3	4	
Q1	1	16	46	33	0	1	10	7	2	3	10	6	0.25
Q2	14	11	51	20	1	2	9	6	3	5	10	3	0.57
Q3	1	9	47	39	0	3	8	7	1	6	12	2	0.06*
Q4	1	5	54	36	0	0	11	7	1	5	10	5	0.046*
Q5	2	14	48	32	0	1	9	8	2	6	8	5	0.17
Q6	0	4	47	45	0	2	7	9	0	6	10	5	0.006*
Q7	1	10	47	38	0	3	8	7	2	6	9	4	0.054
Q8	1	14	48	33	0	1	9	8	4	2	11	4	0.004*
Q9	0	9	41	46	0	1	6	11	1	6	8	6	0.028*
Q10	2	9	49	36	1	2	4	11	1	6	9	5	0.055

1 = Not all true, 2 = Hardly true, 3 = Moderately true, 4 = Exactly true. * Significant at the 0.05 level (2-tailed).

Finally, as indicated in Table 7, age was positively corelated with experience as a nurse practitioner (with a correlation coefficient of −0.827). Specifically, 23 (61%) of the 38 nurses that were aged ≥ 40 years had more than 20 years of nursing experience. These 38 nurses comprised 28% of the study sample.

**Table 7:** Self-efficacy scale response, by age (years) of nurses and years of experience as a nurse (*N* = 135).

Correlation between age (years) and years of experience as a nurse		Years of experience as a nurse				Total
		From 0 to 10	From 11 to 20	From 21 to 30	More than 30	
Age in years	From 22 to 26	12	0	0	0	12
	From 27 to 30	21	0	0	0	21
	From 31 to 35	22	8	0	0	30
	From 36 to 40	9	25	0	0	34
	More than 40	0	15	22	1	38
Total		64	48	22	1	135

Correlation coefficient was 0.827 using Spearman’s correlation coefficient test.

## 4 Discussion

This paper reports on the first study conducted in an academic tertiary healthcare setting in KSA with the goal of assessing nurses’ self-efficacy during the COVID-19 pandemic. The mean GSE score of 31.8 (*SD* = 7.46) indicates a high level of ICU nurses’ self-efficacy during the pandemic despite the associated pressures. A previous cross-sectional study conducted in Spain, involving 308 ICU nurses, corroborated the results of the current study, revealing that ICU nurses reposted high level of self-efficacy (24). In addition, a high level of self-efficacy was correlated with reduced level of stress and enhanced resilience during the COVID-19 pandemic (24). The findings of the present study also align with a recent prospective study conducted in Spain involving 129 ICU nurses (23). Gil-Almagro et al.’s (23) study highlighted the importance of self-efficacy, as a belief in one’s ability to successfully perform specific tasks, as a crucial attribute to enhance clinical performance among ICU nurses during crisis situations such as the COVID-19 pandemic. Enhancing self-efficacy enables ICU nurses to effectively cope with work-related stress, thereby mitigating the onset of anxiety in the short term and reducing the risk of emotional exhaustion in the long term (23).

Although the mean self-efficacy score was reported, the standard deviation reveals variability, indicating that some nurses had significantly lower self-efficacy levels. This suggests diverse experiences that could impact patient care and are not fully represented by the average score. The study findings also suggest that self-efficacy varied significantly based on demographic factors such as age, nationality, and religion. However, the findings revealed no significant differences concerning other important variables like education, nursing experience, or exposure to COVID-19 patients, suggesting that other relevant factors influencing self-efficacy might not have been adequately explored.

Given the paucity of research related to nurses’ self-efficacy in the Arab Gulf region, the findings yielded by this study should be of value to HCW managers in the KSA and other countries where multinational foreign workers make up the bulk of nursing staff. In particular, owing to the growing trend toward the nationalization of various professions (including HCWs) in the KSA, the results reported here point to the urgent need for intensive mentoring and training to ensure that Saudi ICU nurses can deliver the highest level of patient care even under difficult circumstances. Staff placements and rotations should take requirement this into consideration as the younger Saudi ICU nurses will benefit from observing experienced foreign nurses that demonstrate high self-efficacy. The study results also confirm the need for further investigations into the strategies that can be adopted to enhance Saudi nurses’ capabilities as the nation marches towards its Vision 2030 goals.

The current study offers a thorough examination of ICU nurses’ self-efficacy during the COVID-19 pandemic. However, certain limitations must be acknowledged when interpreting the findings presented here, one of which is a relatively small cohort recruited from a single healthcare facility. This study design may hinder the generalizability of the results to the broader nursing population within the country. Future studies should include other frontliner nurses in the study setting as well as those in other government and private hospitals in the region and at the national level to guarantee sufficient representation and generalizability of findings. In addition, the cross-sectional nature of the study precluded the exploration of causal relationships. There could be unaccounted confounding variables or factors influencing the self-efficacy of ICU nurses. A longitudinal study is essential to investigate the progression of self-efficacy over time and to establish causal relationships. Moreover, data on ICU nurses with laboratory-confirmed COVID-19 infection and potential nosocomial acquisition were not collected. Finally, the study did not take into account potential external factors affecting self-efficacy, such as workplace environment, institutional support, or mental health issues arising from the pandemic, which could provide a fuller picture of the ICU nurses’ experiences and self-efficacy. Nonetheless, the significance of this study in addressing the pressing issue of ICU nurses’ self-efficacy during public health crises such as the COVID-19 pandemic should not be overlooked.

## 5 Conclusion

During the COVID-19 pandemic, ICU nurses reported a high level of self-efficacy but significantly varied across their sociodemographic characteristics, specifically age, nationality, and religion. Specifically, older and more experienced nurses demonstrated high self-efficacy. The study implies that as most of the study respondents were expatriates (reflecting the socio-demographics in the Saudi healthcare sector), it is evident that, for the success of the ongoing Saudization program, Saudi ICU nurses must be provided extensive training, allowing them to confidently and efficiently take more independent clinical roles. Consequently, it could be possible that the types of training and mentorship that would be most effective should thus be examined as a part of future research in this domain to support the success of the program.

## Data Availability

The raw data supporting the conclusions of this article will be made available by the corresponding author, without undue reservation.
